# 改良膨胀萎陷法界定肺段间交界面的精确性评估

**DOI:** 10.3779/j.issn.1009-3419.2020.104.15

**Published:** 2020-06-20

**Authors:** 海星 魏, 燚宁 朱, 琦 王, 亮 陈, 卫兵 吴

**Affiliations:** 210029 南京，南京医科大学第一附属医院胸外科 Department of Thoracic Surgery, the First Affiliated Hospital of Nanjing Medical University, Nanjing 210029, China

**Keywords:** 肺肿瘤, 改良膨胀萎陷法, 三维CT支气管血管成像, 肺段切除术, 扩大肺段切除术, Lung neoplasms, Modified inflation-deflation methods, Three-dimensional computed tomography bronchography and angiography, Segmentectomy, Extended segmentectomy

## Abstract

**背景与目的:**

对于早期肺癌，肺段切除术可以获得和肺叶切除术相同的肿瘤学疗效。肺段间交界面的精准界定是肺段手术的关键。本研究采用“改良膨胀萎陷法”行扩大肺段、亚段切除术治疗肺段间、亚段间磨玻璃结节（ground-glass nodules, GGN），评价“改良膨胀萎陷法”界定肺段交界面的精确性，和对扩大切除确保安全切缘宽度的有效性。

**方法:**

回顾性分析本中心采用扩大肺段、亚段切除术治疗的患者83例。术前三维CT支气管血管成像（three-dimensional computed tomography bronchography and angiography, 3D-CTBA）显示结节累及段间静脉。根据三维重建设计手术，扩大切除结节所属的优势肺段或亚段，无法确定优势肺段、亚段时，选择较为简单的肺段、亚段切除方式。术中切断靶段血管、支气管后采用“改良膨胀萎陷法”确定肺段间或亚段间交界面，应用切割缝合器距离膨胀萎陷交界线2 cm-3 cm扩大切除部分相邻肺段或亚段肺组织。观察标本中膨胀萎陷交界线与结节的关系，测量切缘宽度，收集围术期临床资料。

**结果:**

实施扩大肺段切除术56例，扩大肺亚段切除术27例，肺结节平均直径（0.9±0.3）cm。出现清晰可辨膨胀萎陷交界线79例，交界线形成时间（13.6±6.5）min。解剖标本观察发现，结节累及膨胀萎陷交界线55例，其余24例结节距交界线的最小距离（0.6±0.3）cm，平均切缘宽度（2.1±0.3）cm。无术后30 d死亡和重大并发症。

**结论:**

改良膨胀萎陷法可有效界定肺段间、亚段间交界面，以此为标准可确保扩大肺段、亚段切除术治疗段间、亚段间小肺癌的安全切缘。

## Abstract (en)

**Background and objective:**

For early-stage lung cancer, segmentectomy can get the same oncological benefits as lobectomy. Accurate identification of the intersegmental border is the key to segmentectomy. This study used extended segmentectomy and extended subsegmentectomy to treat lung intersegmental and intersubsegmental ground-glass nodules (GGN) by utilizing modified inflation-deflation methods to distinguish the intersegmental and intersubsegmental borders. The accuracy of modified inflation-deflation methods and the effectiveness of extended resection to guarantee a safe surgical margin were evaluated.

**Methods:**

A retrospective analysis of 83 cases of extended segmentectomy and extended subsegmentectomy was conducted. Preoperative three-dimensional computed tomography bronchography and angiography (3D-CTBA) revealed that nodules were involved in intersegmental or intersubsegmental veins. Based on preoperative three-dimensional reconstruction, the surgery was designed to extendedly remove the dominant lung segment or subsegment with nodules involved. When the dominant lung segment or subsegment could not be identified, the simpler lung segment or subsegment was selected for the resection. After the target vessel and bronchus were cut off during the operation, modified inflation-deflation method was used to determine the border, and a stapler was used to resect the adjacent lung segment or subsegment tissue by 2 cm-3 cm around the inflation-deflation boundary line. Then, the relationship between the inflation-deflation boundary line and the nodule and the width of the surgical margin were measured. Clinical data were collected during the perioperative period.

**Results:**

56 extended segmentectomies and 27 extended subsegmentectomies were performed. The average diameter of pulmonary nodules was (0.9±0.3) cm. There were 79 cases with clearly inflation-deflation boundary lines. The average time needed for the appearance of the lines was (13.6±6.5) min. In 55 cases, the nodules were involved with the inflation-deflation boundary lines. Meanwhile, the remaining 24 cases revealed an average minimum distance of (0.6±0.3) cm between nodules and the boundary lines. The average width of surgical margin was (2.1±0.3) cm in these 79 cases. No deaths or major complications appeared during 30 d after operation.

**Conclusion:**

The modified inflation-deflation method can effectively define the intersegmental and intersubsegmental borders, and guarantee the safe surgical margins of extended segmentectomy and extended subsegmentectomy to treat intersegmental and intersubsegmental small lung tumors.

肺癌是威胁人类健康最常见的恶性肿瘤^[1]^，手术是可切除肿瘤的主要治疗方法。对于直径≤2 cm的磨玻璃密度影（ground-glass opacity, GGO）为主的早期肺癌，大量回顾性文献认为亚肺叶切除可获得和肺叶切除相同的肿瘤学疗效^[2, 3]^，并且具有保护肺功能的优点^[4]^。解剖性肺段切除术是亚肺叶手术的主要术式，存在诸多难点，段间交界面的精准界定是肺段切除术的关键。传统膨胀萎陷法认为在离断靶段支气管后全肺通气，靶段会保持萎陷状态，余肺膨胀形成段间交界。然而靶段支气管离断后，手控气囊膨肺难以精准控制，膨肺压力小则保留段无法完全膨胀，压力大时，由于侧枝通气导致靶段也膨胀，所形成的膨胀萎陷交界线不能准确反映段间交界。本中心在传统膨胀萎陷法基础上提出“改良膨胀萎陷法”界定段间交界面^[5]^，即在离断靶段支气管、血管后，术侧肺全肺膨胀，等待一段时间后，靶段膨胀而余肺萎陷形成清晰可见的段间交界。

扩大肺段或亚段切除术是治疗肺段间或亚段间结节的常用方法，为获得充足的切缘，肺段间、亚段间交界面的界定准确性非常重要。肺段间静脉行走在相邻肺段之间，回流相邻肺段的静脉血流，是肺段交界的客观分界线。本研究应用改良膨胀萎陷法，结合术前三维CT支气管血管成像（three-dimensional computed tomography bronchography and angiography, 3D-CTBA）和术中识别行扩大肺段、肺亚段切除术治疗肺段间、亚段间结节，以此评估改良膨胀萎陷法界定段间交界面的精确性和对扩大切除确保安全切缘宽度的有效性。

## 1 资料和方法

### 1.1 临床资料

回顾性分析于南京医科大学第一附属医院胸外科2014年12月-2019年11月行扩大肺段、亚段切除术的83例患者的资料，其中男26例，女57例，年龄27岁-78岁。入组标准：位于肺实质外周1/3的磨玻璃状结节（ground-glass nodules, GGN），直径≤2 cm, 结节内GGO≥50%。所有患者术前均行肺结节CT血管造影（computed tomography angiography, CTA）检查，应用“DeepInsight”软件行3D-CTBA重建，显示结节累及段间静脉。根据三维重建模拟手术路径确定切除范围，扩大切除结节所属的优势肺段或亚段，无法确定优势肺段、亚段时，选择较为简单的肺段、亚段切除方式。手术质控标准：确保切除肺段标本切缘宽度≥2 cm，或切缘宽度≥肿瘤最大径。

### 1.2 手术操作

患者全身麻醉后，双腔气管插管，健侧卧位，健肺通气。胸腔镜途径，采用三孔、两孔或单孔法。根据术者经验，部分患者术前采用CT引导Hook-wire穿刺联合亚甲蓝染色定位结节。在术前3D-CTBA重建指导下，应用“锥式肺段切除术”技术解剖性分离切断靶段支气管、动脉和靶段内静脉，保留段间静脉^[6]^。应用改良膨胀萎陷法界定肺段间、亚段间交界面，即麻醉师吸痰后恢复双肺通气，纯氧膨肺，手动控制气道压维持在15 cmH_2_O-20 cmH_2_O至术侧肺完全膨胀。随后健侧肺单肺通气。5 min-15 min后，保留肺段、亚段完全萎陷呈暗红色，待切除靶段部分膨胀呈粉红色。膨胀的靶段与周围萎陷的肺段、亚段在胸膜表面形成清晰可见的不规则边界，在肺实质内即为肺段间、亚段间的交界面。应用“stapler tailoring”法（缝合器裁剪法）在距离膨胀萎陷交界线2 cm-3 cm扩大切除部分相邻肺段或亚段肺组织^[7]^（图 1）。严重肺气肿、肺纤维化等原因，导致膨胀萎陷交界线不清晰的患者，术中根据段间静脉为段间标志，进行扩大切除。

**1:**
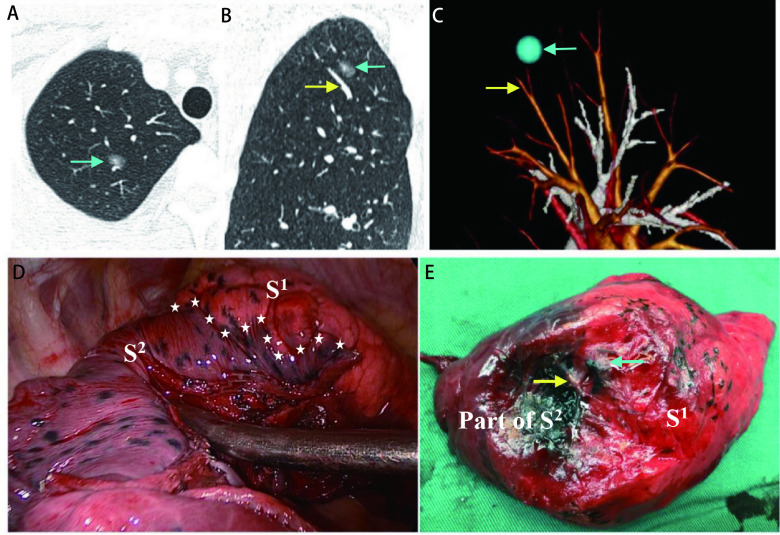
研究流程示意图。以右上肺扩大尖段（S^1^）切除为例。A：CT横断面显示右上肺一直径0.9 cm的GGO为主肺结节（蓝色箭头所示）；B：矢状位重建示，结节紧邻静脉（黄色箭头所示），位置较深，不适合楔形切除；C：3D-CTBA重建肺血管、支气管，显示结节紧邻静脉（黄色箭头所示）为右上肺尖段（S^1^）和后段（S^2^）的段间静脉（V^2^a），结节位于S^1^和S^2^交界处，S^1^为结节所属优势肺段，拟行扩大右肺S^1^切除术；D：改良膨胀萎陷法显示S^1^、S^2^交界线（白色星号线所示）后，距交界线2 cm扩大切除相邻S^2^部分肺组织；E：沿膨胀萎陷交界线切开标本，显示膨胀萎陷交界面，段间静脉（黄色箭头所示）位于交界面内，肺结节（蓝色箭头所示）紧邻段间静脉。 Flow diagram of the study. Take the extended S^1^ of right upper lobe resection as an example. A: The CT cross-sectional view of the right upper lung displayed a GGO nodule with the diameter of 0.9 cm (shown by the blue arrow); B: The sagittal reconstruction of the lung showed that the nodule was close to the vein (the yellow arrow) and was too deep for wedge resection; C: 3D-CTBA revealed that the vein (the yellow arrow) which the nodule was adjacent to was the intersegmental vein (V^2^a) of the S^1^ and S^2^. The operation was designed to extendedly remove the S^1^ with the GGN involved; D: After the boundary line between the S^1^ and S^2^ (shown by the white star line) was defined by the modified inflation-deflation method, resected the adjacent part tissue of S^2^ by 2 cm around the boundary line; E: The specimen was cut along the inflation-deflation boundary line to show the inflation-deflation border. The intersegmental vein (the yellow arrow) was located within the border, and the nodule (the blue arrow) was adjacent to the intersegmental vein. CT: computed tomography; GGO: ground-glass opacity; 3D-CTBA: three-dimensional computed tomography bronchography and angiography; GGN: ground-glass nodules.

胸腔注入温灭菌水测漏。在明显漏气的创面覆盖生物组织材料，喷洒生物蛋白胶。应用“改良式双胸管引流”，即在腋前线主操作孔置入一根16 F乳胶管，外接负压吸引球引流；腋中线胸腔镜孔放置一根24 F胸管引流^[8]^。

入组患者切除标本术中均行冰冻切片病理检查。恶行病例行肺内、纵隔淋巴结采样。肺结节冰冻病理为浸润性腺癌，行系统性淋巴结清扫。采样淋巴结为癌转移，改行肺叶切除术+系统性淋巴结清扫术。

### 1.3 统计分析

使用SPSS 17.0统计软件对统计结果进行分析。收集入组患者围手术期资料。测量标本中结节距离膨胀萎陷交界线的最短距离，记录标本中的切缘宽度、结节位置、病理诊断、肿瘤原发灶-淋巴结-转移（tumor-node-metastasis, TNM）分期。

## 2 结果

应用改良膨胀萎陷法行扩大肺段切除术56例，扩大肺亚段切除术27例（表 1）。病灶位于右上肺29例，右下肺18例，左上肺20例，左下肺16例。采用术前CT引导穿刺定位34例，免穿刺定位49例。术中出现可辨膨胀萎陷交界线79例，占入组患者95.2%。肺结节平均直径（0.9±0.3）cm。术后常规病理示良性病变5例，原位腺癌12例，微浸润腺癌34例，浸润性腺癌31例，肺部转移癌1例。平均切缘宽度（2.1±0.4）cm，平均淋巴结切除数目（4.5±3.1）个，组数（3.4±1.7），均为阴性。无术后30 d死亡和重大并发症（表 2）。

**1:** 扩大肺段、亚段切除术手术方式 Features of extended segmentectomies and extended subsegmentectomies

Characteristics	Different lung	Surgical options	*n*
Extended segmentectomies (*n*=56)			
	Right	S^1^	5
		S^2^	9
		S^3^	5
		S^6^	11
		S^8^	1
		S^8^+S^9^	2
	Left	S^1+2^	1
		S^3^	2
		S^4^+S^5^	7
		S^6^	9
		S^8^	2
		S^9^+S^10^	2
Extended subsegmentectomies (*n*=27)			
	Right	S^1^a	2
		S^1^b	3
		S^2^a	1
		S^2^b	2
		S^3^a	1
		S^3^b	1
		S^8^a	3
		S^9^a	1
	Left	S^1+2^c	6
		S^3^b	3
		S^3^c	1
		S^8^a	3

**2:** 扩大肺段、亚段切除术临床特征 Clinical features of extended segmentectomies and extended subsegmentectomies

Clinical features	
Age (Mean±SD, yr)	52.4±11.0
Gender (*n*)	
Male	26
Female	57
Localization of GGN (*n*)	
Marking with CT-guided methods	34
No marking	49
Nodule size (Mean±SD, cm)	0.9±0.3
Surgical margin (Mean±SD, cm)	2.1±0.4
Different lung (n)	
Right upper lobe	29
Right lower lobe	18
Left upper lobe	20
Left lower lobe	16
Pathological type (*n*)	
Benign	5
Adenocarcinoma in situ	12
Minimally invasive adenocarcinoma	34
Invasive adenocarcinoma	31
Metastasis tumor	1
TNM stages (UICC 8^th^) (*n*)	
0 (Tis）	12
Ⅰa1 (T1aN0M0)	45
Ⅰa2 (T1bN0M0)	20
TNM: tumor-node-metastasis; UICC: Union for International Cancer Control.	

在出现可辨膨胀萎陷交界线的标本中，解剖观察发现，结节累及膨胀萎陷交界线55例，其余24例结节距交界线的最小距离（0.6±0.3）cm，平均切缘宽度（2.1±0.3）cm。出现可辨膨胀萎陷交界线的时间（13.6±6.5）min。

## 3 讨论

GGO为主（GGO成分≥50%）的早期肺癌恶性程度较低，鲜有淋巴结转移，预后及生存较好。对于直径≤2 cm的GGO为主的早期肺癌，大量回顾性文献认为亚肺叶切除可获得和肺叶切除相同的肿瘤学疗效。目前亚肺叶切除的主要术式为楔形切除术和肺段切除术。肺段切除术为解剖性切除，较楔形切除术具有肺内淋巴结采样和更宽大切缘的优点^[9, 10]^。而对于GGO为主的亚厘米肺癌，在保证切缘的前提下两者肿瘤学疗效并无差异^[11, 12]^。楔形切除术和肺段切除术手术方式的选择依据之一是：结节在肺实质所处的位置^[13]^，在确保安全切缘前提下，位于浅表的GGN可选择楔形切除术，位置较深的选择肺段切除术；位于肺段或亚段中央的GGN选择解剖性肺段或亚段切除术，位于肺段或亚段交界的可选择联合亚段切除术或扩大的肺段、亚段切除术。联合亚段切除术技术要求高，难度大，所以对于位置较深的肺段、亚段交界结节，在确保安全切缘前提下扩大的肺段、亚段切除术也是可选方案之一。

肺段切除术作为精准外科手术之一，其基础是精准解剖。但由于肺的支气管、血管变异较多，解剖繁杂，且肺段之间没有明确界限。施行解剖性肺段切除手术存在重多难点。其中段间交界面的精准界定是精准肺段手术的关键^[14]^。交界面的精准界定可以为肺段间的裁剪提供指引，实现肺段解剖性切除。段间交界面不精准，靶段切除不足对于深部肺癌结节极有可能会造成安全切缘不足，影响肿瘤学疗效。相邻肺段切除过多会破坏余肺形态，导致余肺通气、换气功能受影响，造成肺功能不必要的损失。

肺内静脉是解剖性肺段切除术的重要解剖标志。肺段间静脉走形于相邻肺段之间，回流相邻肺段的静脉血，是肺段间边界的天然分界线。同理亚段间静脉走形于相邻亚段之间，是亚段间分界线。本研究利用肺内静脉的解剖学原理，在3D-CTBA重建图像上根据结节与静脉的关系确定结节是否为段间或亚段间结节。应用改良膨胀萎陷法界定段间交界面，如果肺结节位于差异性边界内，说明该方法准确。

本次研究应用改良膨胀萎陷法行扩大肺段、亚段切除术治疗肺段间、亚段间结节83例。入组患者术前均行3D-CTBA重建，显示结节累及段间静脉。术中出现可辨膨胀萎陷交界线79例，术后解剖标本，观测显示段间结节累及膨胀萎陷交界线55例，其余24例结节距交界线的最小距离（0.6±0.3）cm，平均切缘宽度（2.1±0.3）cm。说明改良膨胀萎陷法能够精准界定段间交界面，且此方法界定的段间交界面维持时间久，为精准解剖性分离段间平面提供充足时间。同时此方法可以确保扩大肺段、亚段切除术治疗段间早期肺癌的安全切缘。此方法离断靶段支气管、血管后，纯氧膨肺至全肺完全膨胀，健侧肺单肺通气稍作等待后即可出现精准段间交界面，交界面持续时间长，便于精准分离段间交界面，操作安全、简便、重复性好，值得在临床推广应用。

本中心在开展肺段切除术治疗GGN的早期阶段，术前常规采用CT引导下Hook-wire穿刺定位结节。随着经验积累，对3D-CTBA应用越来越成熟，术前规划非常精准，鉴于穿刺定位的有创性，大部分病例采用免穿刺定位。本次研究中59.0%患者采用了免穿刺定位，免穿刺定位就是基于改良膨胀萎陷法的精确性，不再依赖穿刺针的引导，完全以膨胀萎陷交界线作为段间、亚段间交界的依据。由于严重肺气肿等原因，4.8%的患者无法出现清晰可辨的膨胀萎陷交界线，术中以段间静脉为标志行扩大切除术，还可以结合其他方法精准界定段间交界面。
